# Supplementary material for: Cross-Population Joint Analysis of eQTLs: Fine Mapping and Functional Annotation
Source: PLoS Genet. 2015 Apr 23;11(4):e1005176. doi: 10.1371/journal.pgen.1005176 (PMC4408026; doi:10.1371/journal.pgen.1005176)
Supplement: S1 Text — (PDF) [file pgen.1005176.s001.pdf]

Supplementary Text S1 for "Cross-population Joint Analysis of eQTLs: Fine Mapping and Functional Annotation"

S.1 MCMC Algorithm for Mapping Multiple *cis*-eQTLs

We implemented a Metropolis-Hastings algorithm to perform posterior sampling based on equation (2.3) in the main text. The algorithm is mostly straightforward. To help the Markov chain achieve fast mixing, we implemented a novel proposal distribution based on the result of conditional analysis of multiple *cis*-eQTLs.

We propose two types of simple "local" moves in the MCMC simulations:

1. Change a $\boldsymbol{\gamma}_j$ value for SNP j
2. Swap the values of $\boldsymbol{\gamma}_j$ and $\boldsymbol{\gamma}_k$, for SNPs j and k

where each SNP j is proposed according to a pre-calculated weight w_j. The novelty of the proposal distribution is that we construct the weights w_j's based on the conditional analysis results. More specifically, we start by computing Bayes factors for each *cis*-SNP in a single SNP analysis, and compute a quantity

$$p_j^{(1)} = \frac{\text{BF}_j}{\sum_j^p \text{BF}_j}. \tag{.1}$$

(Note that $p_j^{(1)}$ is proportional to the PIP for SNP j assuming only one eQTL in the *cis* region and a uniform prior inclusion probability). We then find the SNP with the maximum $p_j^{(1)}$ value, say SNP k. In the next round, we control for the genotype of SNP k and repeat the single SNP analysis to obtain $p_j^{(2)}$, which mimics the conditional analysis of secondary *cis*-eQTL signals. Note that SNP k and the SNPs in LD will have single SNP Bayes factor close to 1 in this round. We again add the SNP with the maximum $p_j^{(2)}$ value into the control set. We repeat this procedure, with one additional SNP added into the control set in each round, until the maximum single SNP Bayes factor falls below a pre-defined threshold (we use 10 in practice). Suppose that the procedure ends in t iterations, we then compute the weight for each SNP using

$$w_j = \sum_{r=1}^{t} \theta_r p_j^{(r)} + \theta_{t+1} \frac{1}{p}, \tag{.2}$$

where the sequence $\theta_1, ..., \theta_{t+1}$ forms a decreasing geometric series summing up to 1. The trailing $\frac{1}{p}$ term

in the weight calculation represents a uniform distribution on candidate *cis*-SNPs.

This particular proposal distribution is an extension of what is used in [1], and should be credited to Matthew Stephens (personal communication). Its theoretical backend is related to *sure-independence screening* proposed by [2] in variable selection context.

S.2 Maximum Likelihood Inference of Enrichment Parameters

This section gives the technical details of MCMC-within-EM algorithm. Given the hierarchical model described in the main text, we are interested in performing maximum likelihood inference of enrichment parameter $\boldsymbol{\alpha}$. Treating $\{\boldsymbol{\Gamma}^1, ...\boldsymbol{\Gamma}^q\}$ across all q genes as missing data, the complete data likelihood can be written as

$$P(\{\boldsymbol{Y}^g\}, \{\boldsymbol{\Gamma}^g\} \mid \{\boldsymbol{G}^g\}, \{\boldsymbol{D}^g\}, \boldsymbol{\alpha}) = \prod_{g=1}^{q} P(\boldsymbol{\Gamma}^g \mid \boldsymbol{D}^g, \boldsymbol{\alpha}) \cdot \prod_{g=1}^{q} P(\boldsymbol{Y}^g | \boldsymbol{\Gamma}^g, \boldsymbol{G}^g). \tag{.3}$$

We apply an EM algorithm to find the MLE of $\boldsymbol{\alpha}$. Because vector $\boldsymbol{\gamma}_j^g$ only takes values in $\{\mathbf{0}, \mathbf{1}\}$, using a loose notation, we represent vectors $\mathbf{0}$ and $\mathbf{1}$ with the corresponding binary scalar values. It then follows that

$$P(\boldsymbol{\Gamma}^g \mid \boldsymbol{D}^g, \boldsymbol{\alpha}) = \prod_j \left[\left(\frac{\exp(\boldsymbol{\alpha}'\boldsymbol{\delta}_j^g)}{1 + \exp(\boldsymbol{\alpha}'\boldsymbol{\delta}_j^g)} \right)^{\boldsymbol{\gamma}_j^g} \left(\frac{1}{1 + \exp(\boldsymbol{\alpha}'\boldsymbol{\delta}_j^g)} \right)^{1-\boldsymbol{\gamma}_j^g} \right]. \tag{.4}$$

The complete data log-likelihood is given by

$$\begin{aligned} \log L(\boldsymbol{\alpha}; \{\boldsymbol{Y}^g\}, \{\boldsymbol{\Gamma}^g\}, \{\boldsymbol{G}^g\}, \{\boldsymbol{D}^g\}) = & \sum_{g=1}^{q} \sum_{j=1}^{p_g} \boldsymbol{\gamma}_j^g (\boldsymbol{\alpha}'\boldsymbol{\delta}_j^g) - \sum_{g=1}^{q} \sum_{j=1}^{p_g} \log[1 + \exp(\boldsymbol{\alpha}'\boldsymbol{\delta}_j^g)] \\ & + \sum_{g=1}^{q} \log[P(\boldsymbol{Y}^g | \boldsymbol{\Gamma}^g, \boldsymbol{G}^g)] \end{aligned} \tag{.5}$$

The EM algorithm initiates by an arbitrary value of $\boldsymbol{\alpha}$, namely, $\boldsymbol{\alpha}^{(1)}$. In the E-step of t-th iteration, we compute

$$\begin{aligned} \mathrm{E}[\log L(\boldsymbol{\alpha}\,; \{\boldsymbol{Y}^g\}, \{\boldsymbol{\Gamma}^g\}, \{\boldsymbol{G}^g\}, \{\boldsymbol{D}^g\}) \mid \{\boldsymbol{Y}^g\}, \{\boldsymbol{G}^g\}, \boldsymbol{\alpha}^{(t)}] = & \sum_{g=1}^{q} \sum_{j=1}^{p_g} \mathrm{E}\left(\boldsymbol{\gamma}_j^g \mid \boldsymbol{Y}^g, \boldsymbol{G}^g, \boldsymbol{\alpha}^{(t)}\right) (\boldsymbol{\alpha}'\boldsymbol{\delta}_j^g) \\ - \sum_{g=1}^{q} \sum_{j=1}^{p_g} \log[1 + \exp(\boldsymbol{\alpha}'\boldsymbol{\delta}_j^g)] + \sum_{g=1}^{q} \mathrm{E}\left(\log[P(\boldsymbol{Y}^g | \boldsymbol{\Gamma}^g, \boldsymbol{G}^g)] \mid \boldsymbol{Y}^g, \boldsymbol{G}^g, \boldsymbol{\alpha}^{(t)}\right) & \end{aligned} \tag{.6}$$

Note that the last term does not contain parameter $\boldsymbol{\alpha}$. In the M-step of the t-th iteration, we find

$$\boldsymbol{\alpha}^{(t+1)} = \arg\max_{\boldsymbol{\alpha}} \left(\sum_{g=1}^{q} \sum_{j=1}^{p_g} \mathrm{E}\left(\boldsymbol{\gamma}_j^g \,|\, \boldsymbol{Y}^g, \boldsymbol{G}^g, \boldsymbol{\alpha}^{(t)} \right) (\boldsymbol{\alpha}' \boldsymbol{\delta}_j^g) - \sum_{g=1}^{q} \sum_{j=1}^{p_g} \log[1 + \exp(\boldsymbol{\alpha}' \boldsymbol{\delta}_j^g)] \right) \tag{.7}$$

The objective function in (.7) coincides with the log-likelihood function of a logsitic regression model treating each gene-SNP pair as an independent observation, however with the usual binary response variable replaced by the conditional expectations. By this connection, the maximization step can be carried out by fitting the corresponding modified logistic regression model treating conditional expectations as responses (i.e., via an iterative re-weighted least square algorithm). This also implies that in the E-step, it is only required to compute $\mathrm{E}(\left(\boldsymbol{\gamma}_j^g \,|\, \boldsymbol{Y}^g, \boldsymbol{G}^g, \boldsymbol{\alpha}^{(t)}\right) = \Pr(\boldsymbol{\gamma}_j^g = 1 \,|\, \boldsymbol{Y}^g, \boldsymbol{G}^g, \boldsymbol{\alpha}^{(t)})$, i.e., the PIP for each gene-SNP pair, which we obtain from the MCMC sampling.

To summarize, we outline the procedure of the MCMC-within-EM algorithm based on the above derivation as follows

1. At $t = 1$, initiate $\boldsymbol{\alpha} = \boldsymbol{\alpha}^{(1)}$
2. Compute prior $\Pr(\boldsymbol{\Gamma}^g \mid \boldsymbol{D}^g, \boldsymbol{\alpha}^{(t)})$, and run MCMC algorithm for multiple *cis*-eQTL analysis for each gene g
3. Compute $\Pr(\boldsymbol{\gamma}_j^g = 1 \,|\, \boldsymbol{Y}^g, \boldsymbol{G}^g, \boldsymbol{\alpha}^{(t)})$ for each gene-SNP pair from the posterior samples
4. Find $\boldsymbol{\alpha}^{(t+1)}$ by fitting a logistic regression model treating $\Pr(\boldsymbol{\gamma}_j^g = 1 \,|\, \boldsymbol{Y}^g, \boldsymbol{G}^g, \boldsymbol{\alpha}^{(t)})$ as response variable and $\{\boldsymbol{D}^g\}$ as observed covariates
5. Repeat 2 to 4 until convergence

S.3 Single Base-pair Resolution Annotation of Genetic Variants Predicted to Affect Transcription Factor Binding

The approach and validation of the annotation are detailed in [3] and here we provide a summary for the annotation method. The method used to develop the annotation is based on the CENTIPEDE approach that can predict TF activity from integrating sequence motifs together with functional genomics data. This approach gains the most information from high-resolution data such as DNase-seq or ATAC-seq [4]. In the original CENTIPEDE approach, the sequence models are pre-determined; e.g, k-mers

or previously defined position weight matrix (PWM) models from databases such as TRANSFAC and JASPAR. However, many motif models were created with very few sample sequences obtained from known TF binding sites and do not represent the full spectrum of sequence variation that can be tolerated without affecting binding. To better capture this range, it is necessary to include motif instances that may not be a perfect match to the original PWM, but have evidence of binding in the human genome. In [3] , we introduced a novel approach extending CENTIPEDE to re-adjust the sequence model for TF binding using only DNase-seq data in two steps:

Step 1: Initial CENTIPEDE scan and motif recalibration. After scanning the genome for motif matches (using 1949 seed motifs), we extracted DNase-seq data at these sites using 653 samples publicly available from the ENCODE and Roadmap Epigenomics projects. For each motif and only for this initial step, we used a reduced subset of motif matches that include the top 5,000 instances on the human genome; and up to 10,000 additional sequences in the human genome that do not have a high score. The low scoring motif instances were chosen from human sequences that have orthologous high scoring motif instances in the chimp or rhesus genome. We then applied the CENTIPEDE model to survey TF activity for each 1,272,697 tissue-TF pair. For each pair we then determined that the TF is active if the motifs instances that exhibit a CENTIPEDE footprint can be predicted from the PWM score (z-score > 5). Using this criterion, we determined that 1,891 TF motifs are active in at least one tissue. We then recalibrated the PWM model for each active motif using the sequences of all motif matches that have a DNase-seq footprint (CENTIPEDE posterior >0.99). Using this procedure, the probabilities of certain bases are readjusted, but the core part of the motif and its consensus sequence is largely maintained.

Step 2: Full genome CENTIPEDE scan and genetic variant analysis. Using these newly updated sequence models we scanned the human genome for all possible matches both to the reference and to alternate alleles from genetic variants cataloged in the 1000 Genomes (1KG) Project [5] and used the CENTIPEDE algorithm to assess the probability that each motif instance is bound by a TF. In this second step, we included all high and low scoring PWM matches down to a CENTIPEDE prior probability of binding of 10%. In this paper we focus only on SNPs found in CENTIPEDE footprints discovered in LCLs with a posterior probability > 0.99. In total about 600,000 SNPs are in LCL footprints, of which about half are predicted to strongly affect binding, affecting the prior odds of binding $\geq$ 20-fold (binding variants) based on the logistic sequence model hyper prior in the CENTIPEDE model.

S.4 Automatic Clustering of Independent eQTL Signals from MCMC Output

We designed a hierarchical clustering based algorithm to automatically parse the MCMC output and recognize potentially multiple independent association signal clusters.

Let $\boldsymbol{M}$ denote the set of posterior models sampled by the MCMC algorithm. For each model $m_k \in \boldsymbol{M}$, we denote its posterior model probability by p_{m_k}. We define a "distance" between SNP i and SNP j by

$$d_{ij} = \sum_{m_k \in \boldsymbol{M}} p_{m_k} \mathbf{1}\left\{i \in m_k,\, j \in m_k,\, i \neq j\right\}.$$

The above definition is the key to the algorithm. Every SNP has distance 0 to itself. For SNPs with high LD, they are inter-changeable of each other but almost never co-exist in a single posterior model, and consequently those SNPs have distances ≈ 0 with each other. On the other hand, SNPs representing independent signals do often co-exist in posterior models and have non-zero distance between each other. Consider a simple example with 3 SNPs: SNP 1 alone represents an independent signal and appears in all posterior models, SNP 2 and 3 are in high LD and jointly represent another independent signal. Suppose that from the posterior sampling, we observe posterior model $[1, 2]$ and $[1, 3]$ 40% and 60% of the time, respectively. The resulting pair-wise "distance" matrix based on our definition is then given by

$$\begin{pmatrix} 0.0 & 0.4 & 0.6 \\ 0.4 & 0.0 & 0.0 \\ 0.6 & 0.0 & 0.0 \end{pmatrix}$$

We then perform a hierarchical clustering based on the resulting pair-wise "distance" matrix constructed from the MCMC samples. By default, we choose the cluster number to be the maximum model size observed in all the posterior models. Based on the clustering result, we compute a cluster-level PIP by summing over the SNP-level PIPs within each inferred cluster. In the above toy example, by selecting cluster number $K = 2$, SNP 1 forms a cluster with the cluster-level PIP $= 1.0$ and SNP 2 and 3 form another cluster with cluster-level PIP $= 1.0$ as well.

It should be noted that our pair-wise distance measure is very similar to the commonly used Kullback-Liebler distance in measuring the independence between a pair of SNPs. However, our measure is more convenient to compute from the posterior model probabilities in the MCMC output. Neither our measure or the Kullback-Liebler distance is technically a well-defined distance metric. However in practice, the

clustering algorithm works well with these pseudo distance measures.

We find that this algorithm performs reasonably well in practice. One of the most obvious advantage is that it automatically groups SNPs in high LD and recognizes clusters with high PIPs at cluster level. Nevertheless, we still view this algorithm as a heuristic tool to simply aid the post-hoc analysis. In addition, we find that checking the LD patterns from the genotype data for each inferred cluster can serve as a useful independent validation.

S.5 Additional Details of Simulation Studies

In this section, we provide additional details on generating and analyzing simulated expression-genotype data in our simulation studies.

In the main text, we have detailed the procedure to assemble the genotype data from the GUEVADIS project. For each of the 1,500 simulated genes, we randomly sampled 1, 2, 3 or 4 regions to harbor a causal eQTL with probabilities $0.40, 0.30, 0.20$ and 0.10, respectively. (For example, with probability 0.20, the simulated gene contain 3 independent eQTL signal.) Once the number of independent signals was determined, we randomly selected a single causal SNP as the causal SNP from each eQTL region according to a discrete uniform distribution.

For each causal SNP, we simulated its effects in the five populations according to the following scheme. We first generated a mean effect from $\bar{\beta} \sim \mathrm{N}(0, 0.6^2)$, then the eQTL effect of the causal SNP for each population was subsequently drawn from the distribution $\beta \sim \mathrm{N}(\bar{\beta}, \frac{\bar{\beta}^2}{100})$. With this procedure, the resulting eQTL effects are highly correlated across populations however with some low levels of heterogeneity. It is worth pointing out that this generating model is different from the model that we used for the Bayesian analysis. Finally, we generated the expression phenotype separately in each population using the linear model (2.1), with the additional random error vector simulated from $\mathrm{N}(0, \boldsymbol{I})$.

To perform single SNP analysis on the simulated data set, we carried out a fixed effect meta-analysis procedure. More specifically, for SNP j in population group i, we computed a z-score $z_{i,j} = \hat{\beta}_{i,j}/\mathrm{se}(\hat{\beta}_{i,j})$ by fitting a simple linear regression model. We then computed a fixed effect meta-analysis test statistic

$$\bar{Z}_j = \frac{\sum_i w_{i,j} z_{i,j}}{\sum_i w_{i,j}},$$

where the weight is obtained by $w_{i,j} = 1/\mathrm{se}(\hat{\beta}_{i,j})^2$. The variance of $\bar{Z}_j$ is calculated by

$$\mathrm{Var}(\bar{Z}_j) = \frac{1}{\sum_i w_{i,j}}.$$

For each SNP, we computed an overall z-value and obtained its corresponding p-value.

The core procedure for the conditional analysis in each round is similar to the above descried single SNP analysis. However, instead of fitting a simple linear regression model, we fit a multiple regression model controlling for the top associated SNPs identified from the previous rounds. We started the procedure with an empty set of SNPs to be controlled for and halted the procedure until the most significant fixed effect meta-analysis p-value is larger than the pre-defined threshold.

References

1. Guan Y, Stephens M, et al. (2011) Bayesian variable selection regression for genome-wide association studies and other large-scale problems. The Annals of Applied Statistics 5: 1780–1815.

2. Fan J, Lv J (2008) Sure independence screening for ultrahigh dimensional feature space. Journal of the Royal Statistical Society: Series B (Statistical Methodology) 70: 849–911.

3. Moyerbrailean GA, Harvey CT, Kalita CA, Wen X, Luca F, et al. (2014) Are all genetic variants in dnase i sensitivity regions functional? bioRxiv : 007559.

4. Buenrostro JD, Giresi PG, Zaba LC, Chang HY, Greenleaf WJ (2013) Transposition of native chromatin for fast and sensitive epigenomic profiling of open chromatin, dna-binding proteins and nucleosome position. Nature methods .

5. 1000 Genomes Project Consortium, et al. (2012) An integrated map of genetic variation from 1,092 human genomes. Nature 491: 56–65.
