# Supplementary figures and images for: Cross-Population Joint Analysis of eQTLs: Fine Mapping and Functional Annotation
Source: PLoS Genet. 2015 Apr 23;11(4):e1005176. doi: 10.1371/journal.pgen.1005176 (PMC4408026; doi:10.1371/journal.pgen.1005176)

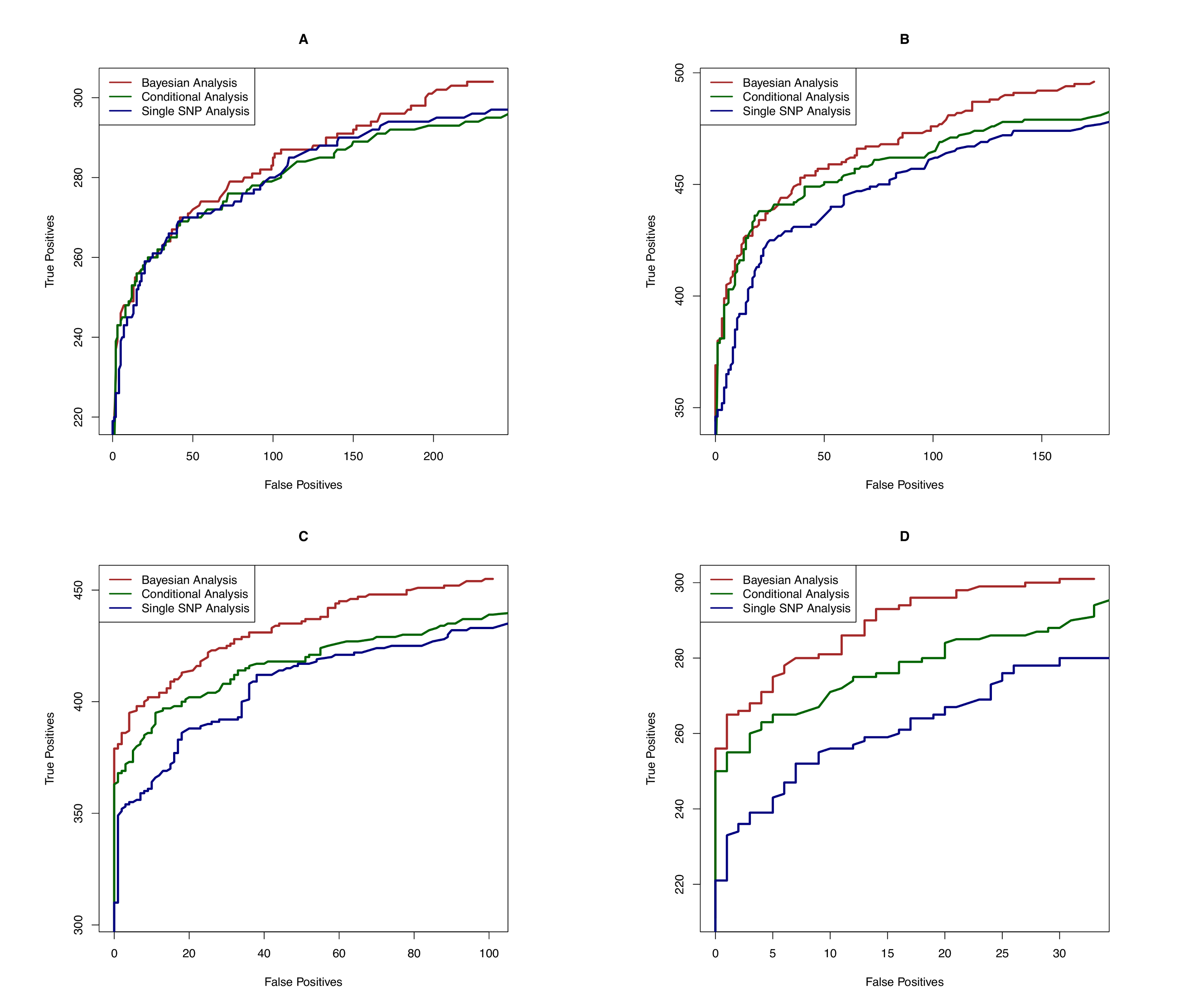

Supplement: S1 Fig — Panels A, B, C and D show simulation results for genes containing 1, 2, 3 and 4 cis-eQTLs, respectively. In each panel, three competing methods are compared: the proposed Bayesian multi-SNP analysis method (brown line), a conditional meta-analysis method (dark green line) and a single SNP meta-analysis approach (navy blue line). Each plotted point on each panel represents the number of true positive findings versus the number of false positive findings of a given method at a particular threshold. In the simulation study, the numbers of genes containing 1, 2, 3 and 4 cis-eQTLs are 579, 457, 307 and 157, respectively. For genes that harbor only a single eQTL, all three methods yield similar performance. However, when there exist more eQTLs in a gene, the proposed Bayesian multi-SNP approach shows superior power over the two competing procedures. (TIFF) [file pgen.1005176.s004.tiff]

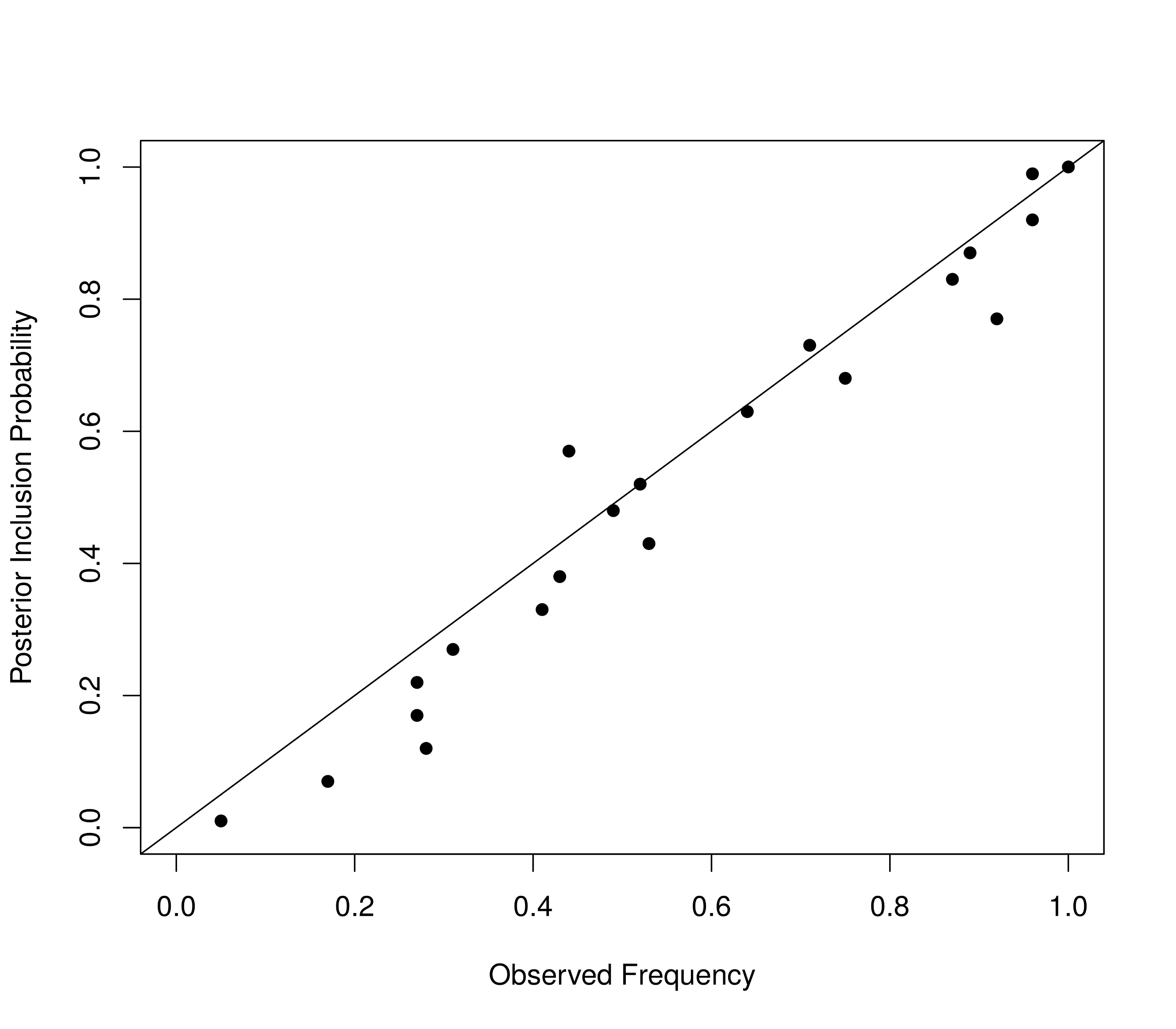

Supplement: S2 Fig — Using the simulated data set, we grouped the reported 1500 × 100 regional PIP values, ranging from 0.0 to 1.0, into 20 bins of width 0.05. For each bin, we plot the mean PIP value and the corresponding frequency of true causal regions represented in the bin. All points are closely located to the diagonal line, indicating that the reported PIPs are relatively well-calibrated. (TIFF) [file pgen.1005176.s005.tiff]

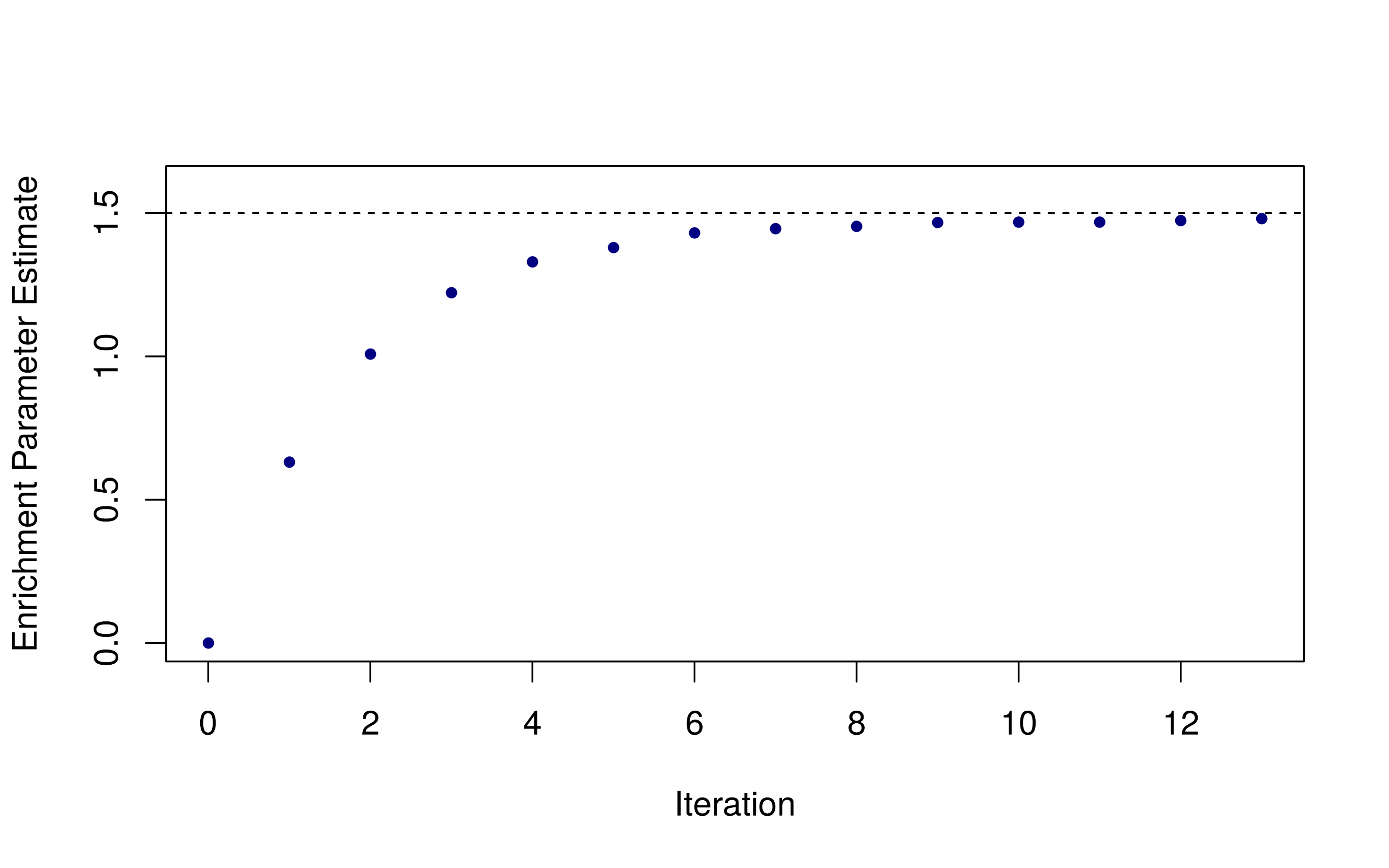

Supplement: S3 Fig — In this simulated data set, the true enrichment parameter is set to 1.50 (the dotted horizontal line). The algorithm initiate the parameter value at 0 and quickly reaches to the close neighborhood of the true value. (TIFF) [file pgen.1005176.s006.tiff]
