# Supplementary material for: Cross-Population Joint Analysis of eQTLs: Fine Mapping and Functional Annotation
Source: PLoS Genet. 2015 Apr 23;11(4):e1005176. doi: 10.1371/journal.pgen.1005176 (PMC4408026; doi:10.1371/journal.pgen.1005176)
Supplement: S1 Table — The estimates are obtained from the hierarchical model described in [14, 21]. For each grid value of ϕ2ϕ2+ω2, ranging from 0.0 to 1.0, we estimate its probability weight by pooling information of all gene-SNP pairs. The grid value 0 indicates a fixed (i.e., the most consistent) eQTL effect across all populations and has the most estimated weight. The grid value 1 indicates completely independent eQTL effects across populations. The results indicate that across population groups, on average, the eQTL effects exhibit low level of heterogeneity. (PDF) [file pgen.1005176.s007.pdf]

Heterogeneity Level ($\frac{\phi^2}{\phi^2+\omega^2}$)	Estimated Weight
0.0	0.651
0.1	0.204
0.2	0.084
0.3	0.035
0.4	0.014
0.5	0.006
0.6	0.003
0.7	0.002
0.8	0.000
0.9	0.000
1.0	0.000
