# Supplementary material for: Cross-Population Joint Analysis of eQTLs: Fine Mapping and Functional Annotation
Source: PLoS Genet. 2015 Apr 23;11(4):e1005176. doi: 10.1371/journal.pgen.1005176 (PMC4408026; doi:10.1371/journal.pgen.1005176)
Supplement: S2 Table — The Bayesian method utilizes PIPs from the multi-SNP eQTL fine mapping analysis. The approach in comparison ranks the SNPs by their single SNP testing p-values in each gene, and classifies the top associated SNPs as the “causal” eQTLs if their p-values pass the significance threshold at FDR 5% level. Both methods (conservatively) control the type I errors at desired 5% level. The PIP based method show much improved powers comparing to the standard approach in all three alternative scenarios where the magnitude of enrichment ranges from small to modest. (PDF) [file pgen.1005176.s008.pdf]

Method	Type I error		Power	
	$\alpha_1 = 0$	$\alpha_1 = 0.25$	$\alpha_1 = 0.50$	$\alpha_1 = 0.75$
Multi-SNP PIP	0.02	0.28	0.62	0.86
p-value Classification	0.01	0.12	0.17	0.33
